# Surgical management of complex brain arteriovenous malformations with hybrid operating technique: study protocol of a prospective registry and a pragmatic clinical trial

**DOI:** 10.1186/s12883-019-1289-3

**Published:** 2019-04-30

**Authors:** Mingze Wang, Yuming Jiao, Yong Cao, Shuo Wang, Jizong Zhao

**Affiliations:** 1 Department of Neurosurgery, Beijing Tiantan Hospital, Capital Medical University, Beijing, 100070 China; 2 China National Clinical Research Center for Neurological Diseases, Beijing, 100070 China; 3 Center of Stroke, Beijing Institute for Brain Disorders, Beijing, 100070 China; 4 Beijing Key Laboratory of Translational Medicine for Cerebrovascular Disease, Beijing, 100070 China

**Keywords:** Brain arteriovenous malformation, Neurosurgery, One-staged hybrid operation, Pragmatic clinical trial, Registry

## Abstract

**Background:**

Complex brain arteriovenous malformations (bAVMs) in ≥3 Spetzler-Martin grades have long been challenges among cerebrovascular diseases. None of the traditional methods, such as microsurgical operation, endovascular intervention, or stereotactic radiotherapy, can completely eliminate complex bAVMs without a risk of neural function deterioration. The multistaged hybrid operation solved part of the challenge but remained risky in the installment procedures and intervals. The one-staged hybrid operation was applied in the surgical treatment of cerebrovascular diseases and proved to be a potentially safe and effective method for curing complex bAVMs. However, lacking the support of high-level evidence, its advantages remain unclear. This study was proposed to validate the benefits and risks of one-staged hybrid operation in the treatment of complex bAVMs, as well as its indications, key technologies, and workflows.

**Methods:**

The study is being conducted from Jan 2016 to Dec 2020 with 20 cooperation centers. It consists of 2 sets. The registry set is designed as a prospective real-world registry. The trial set is designed as a prospective pragmatic clinical trial, specifically for the patients with perforating arterial feeders. The two sets share a common grouping: the traditional operation group and the one-staged hybrid operation group. The assignment is based on the clinical condition in the registry set and is randomized in the trial set. End points will be evaluated at scheduled time points. The safety and efficiency of one-staged hybrid operation in treating complex bAVMs will be validated.

**Discussion:**

The study is designed for a real-world exploration of benefits and risks of one-staged hybrid operation in the treatment of complex bAVMs. The two-set design reduces the compromise of clinical practice due to the study and improves the statistical power and research quality with a practical sample size. In the study, advantages of the one-staged hybrid operation will be evaluated and compared to those of traditional operation. A spanning development of neurosurgical operation might be facilitated by the study, which means a higher cure rate and lower disability rate in patients with complex bAVMs.

**Trial registration:**

The study was retrospectively registered in ClinicalTrials.gov (NCT03774017) on 11th Dec, 2018.

## Background

The surgical treatment of brain arteriovenous malformations (bAVMs) in deep and eloquent locations with complex angioarchitectures has long been a challenge [1–4]. These bAVMs were usually ≥3 in Spetzler-Martin’s grading system. The neurosurgical operation used to be the most effective method of eliminating the bAVMs. With the consideration of neural function protection, it no longer worked, not even with the revolutionary microsurgical techniques [5–11], especially in the complex lesions [12]. On the other hand, endovascular embolization could eliminate the bAVMs directly by obliterating the feeding arteries and nidus without the destruction of local parenchyma [13]. With this feature, the neural function of the brain stem and other eloquence areas were partially preserved [14]. Despite the development of endovascular instruments, materials, and techniques [15–22], limitations of manipulation and prognosis essentially existed in complex bAVMs. Among a variety of suboptimal events, intraoperative bleeding and residue were the most remarkable ones [23, 24]. The cooperation of microsurgery and endovascular intervention typically showed up as several stages of endovascular embolization followed by a scheduled microsurgical resection or radiotherapy (AKA multistaged hybrid operation). It significantly reduced the rates of mortality, neural deficit and postoperative epilepsy in complex bAVMs [25–27]. However, the latent risks of multistaged hybrid operation should be alerted, such as the trap of catheters, intraoperative bleeding, and venous sinus thrombosis in installment treatments, as well as rebleeding, infarction, and epilepsy in intervals [28, 29]. These limitations were not overcome until the one-staged hybrid operation was introduced from cardiac operations [30]. The procedures of microsurgery and endovascular intervention were integrated together in one operation without the transfer of patients. These procedures were applied in the surgical treatment of cerebrovascular diseases and proved to be a potentially safe and effective method of curing complex bAVMs [31, 32].

As an innovative operation mode, no clinical research has been set up to investigate the benefits and risks of the one-staged hybrid operation in the treatment of complex bAVMs as compared to traditional methods. Only a few descriptive studies have been published since it appeared [31–33], and these can hardly be regarded as providing high-level evidence. In addition, indications, key technologies, workflows, etc. remained unclear. We proposed this study to solve the problems above.

The aim of this study is to validate the benefits and risks of the one-staged operating technique in the treatment of complex bAVMs. Operative indications, key technologies, and workflows will be further explored in our study.

## Methods

### Aims

To validate the benefits and risks of one-staged operating technique in the treatment of complex bAVMs, as well as the operative indications, key technologies, and workflows.

### Study design

**Fig. 1:**
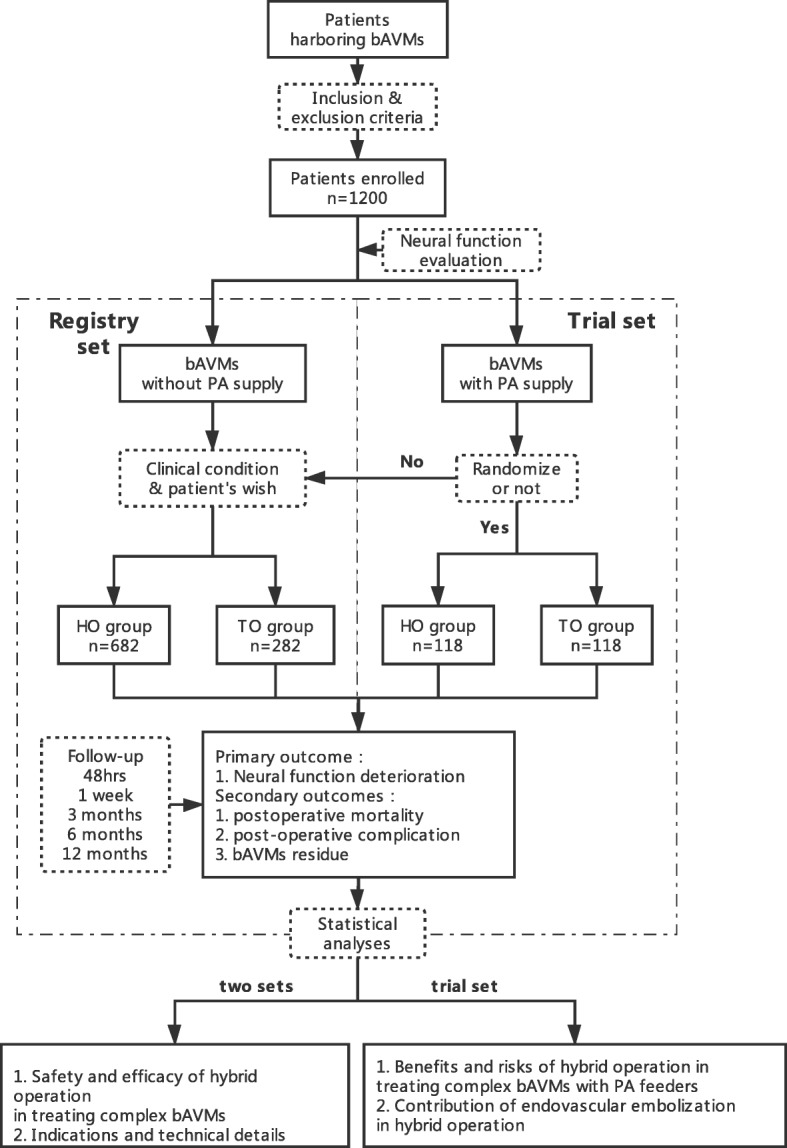
Roadmap of study protocol. *bAVMs* = brain arteriovenous malformations; *PA* = perforating artery; *HO* = hybrid operation; *TO* = traditional operation

The study consists of 2 parts: the registry set and the trial set. The two sets of this study share a common data pool, which contains all the enrolled patients. The registry set is designed as a multicentric, prospective, real-world registry (Research on Hybrid Operation Technique in the Treatment of Complex Brain Arteriovenous Malformations; ClinicalTrials.gov ID: NCT 03774017), and it occupies the whole data pool. The patients involved will receive traditional operation (TO) or one-staged hybrid operation (HO) and will be registered in the corresponding TO group or HO group. End points will be evaluated at scheduled time points. The safety and efficiency of the one-staged hybrid operation in treating complex bAVMs will be validated. The trial set is designed as a multicenter, prospective, pragmatic clinical trial (PCT). Patients in this set will be registered in the registry set in the meantime. PCT is a real-world modification of randomized clinical trial (RCT), consisting of an RCT part and a registry part. Patients have the right to choose the part in which they want to participate. The candidates in this set are patients harboring bAVMs with perforating arterial (PA) feeders. To them, an extra written informed consent is required. Patients who agree to take part in the RCT part will be randomly assigned to the HO or TO group and receive the corresponding treatment. Both of the sets share the same end points. The purpose of the trial set is to validate the benefits and risks of hybrid operation for lesions with perforating arterial feeders and the contribution of endovascular embolization. A strategy of multicenter stratified blocked randomization is used in the central randomization system (CRS), which is contained in Electrical Data Capture (EDC) system. When registering a participant in EDC system in the trial set, CRS generates an assignment result according to the demographic information. Each center is set as a stratifying level, which could reduce the differentiation of sample size between groups. The blind codes, generated by statistician, remain blind during randomizations to avoid potential selection bias. The design of this study is presented in Fig. 1. The grouping assignment is blinded to outcomes assessors. Both sets will be conducted between Jan 2016 and Dec 2020. The study is hosted by Beijing Tiantan Hospital, with the participation of 20 cooperation centers, including academic, military, and other public hospital (Table 1).

**Table 1:** List of Participating Units

No.	Participant Unit	Ethics Approval Number
1	Beijing Tiantan Hospital, Capital Medical University	KY2017–012-02
2	Huashan Hospital, Fudan University	2017–404
3	Xuanwu Hospital, Capital Medical University	[2017]048
4	Affi. 2nd Hospital, Zhejiang University	2016–137
5	Rocket Army General Hospital	KY2016015
6	Qingdao Municipal Hospital	(2016) No.6
7	Qilu Hospital, Shandong Univiersity	KYLL-2019B-002
8	Shanghai No.6 People’s Hospital	YS-2016-113
9	Affi. 2nd Hospital, Suzhou University	JD-LK-2018-060-02
10	Tianjin Huanhu Hospital	2017–5
11	Huaxi Hospital, Sichuan University	2017(No.116)
12	General Hospital of Shenyang Military Area Command	(2016)13
13	Zhongnan Hospital, Wuhan University	2,016,080
14	Tangdu Hospital, the 4th Military Medical University	TDLL-2016500
15	Affi. 2nd Hospital, Kunming Medical University	PJ-2018-12
16	Chongqing Xinan Hospital	KY201765
17	No. 1 Hospital, China Medical University	2016QL013–1
18	Affl. 1st Hospital, Fujian Medical University	KYYS2016–001
19	Nanjing General Hospital of Nanjing Military Area Command	2016NZZDZX-006
20	Chinese PLA General Hospital	S2016–073-01

### Patient and public involvement

The research question originates from the practical problems arising from the publications in recent years. No patient was involved in the development of the research question, outcome measures, or study design. Complex bAVMs could hardly receive a satisfying clinical prognosis with any single method. Although some of the patients are suffering from intractable epilepsy or microhemorrhage, which are considered to be operative indications, the expectant therapy is the most common method. Referring to the present study on one-staged hybrid operation, the advantages of microsurgical and endovascular embolization were significantly integrated as mentioned above. A new therapeutic concept was proposed by this novel technique. Most importantly, patients with complex bAVMs have a chance to be cured. The study is designed to validate the efficiency and safety of the one-staged hybrid operation. With the development of microsurgical skills and life-sustaining technology, neural function deterioration caused the most concern among outcomes. It is the main reason deterring the patients from operation, as well [6, 7, 9, 10]. For this reason, the neural function deterioration is chosen to be the primary outcome in this study. Traditional outcomes, such as postoperative mortality, operation-related complications, and bAVMs residue, are extracted to secondary outcomes as evaluating indexes.

The final results of the study will not be disseminated to the study participants but will be published in research articles. In the beginning of the study, as there was no reference result, the benefits, risks, and possible prognosis were acknowledged in preoperative discussions among neurosurgeons, interventional radiologists, and anesthesiologists before the study participants were informed. After a certain amount of cases are accumulated, the participants will be informed of the latest results as the risk reference.

All patients involved are introduced to the operative process, advantages and shortcomings of one-staged operation and traditional operation. Individualized risks of the two methods are evaluated in the preoperative discussion and announced to the patients. Participants make the selection after weighing the benefits, risks and financial burden of different operative methods. As the majority of medical expenses are covered by the Urban Residents’ Basic Medical Insurance and Rural Cooperative Medical Service for Chinese citizens, there is no extra financial compensation in this study.

### Study participants

Patients harboring bAVMs will be regarded as candidates for the duration of the study. Those candidates will be further screened with inclusion and exclusion criteria by attending physicians in all the participating centers. Patients will be involved after giving their written informed consent. Demographic information (age, gender, race, provenance, occupation) will be recorded as baseline features. Present history, especially the bAVMs-related primary symptom and operation history, will be recorded for the analysis of risk factors, as well as the past history, family history, smoking and drinking history. Morphological and location features of bAVMs will be interpreted by experienced radiologists and surgeons according to the images of cranial computed tomography (CT) and magnetic resonance (MR). Angioarchitecture features (origin and type of feeding arteries, situation of nidus and draining veins) will be specially evaluated by digital subtraction angiography. All image data will be stored in digital imaging and communications in medicine (Dicom) format. Milestones of management (dates of admission, operation, and discharge) will be recorded, and minute units will be used for the starting and finishing time of manipulations during the hybrid operation. Materials used in the endovascular and microsurgical procedures will be finely recorded in their type and quantity. Cases operated by a special manipulating technique will be labeled. The neural conditions and outcomes of participants will be evaluated by physicians using the modified Rankin Scale (mRS) and the National Institute of Health Stroke Scale (NIHSS) at the time of admission, 2 days after operation, 1 week after operation and at the follow-up time points after discharge. All data will be collected by full-time clinical research coordinators (CRCs) with both paper documents and a web-based electronic data capture system (EDC system), and they will be quality-controlled by clinical research associates (CRAs) from a third-party contract research organization (CRO). All participants will be informed, asked for written informed consent, and allowed to withdraw at any time.

### Inclusion criteria

1. patients aged≤70 years old;
2. diagnosed with arteriovenous malformations (AVMs) in brain parenchyma (including cerebrum and cerebellum) by DSA, with/without dura arteriovenous fistula;
3. with any operative indications as follows:
  1. with stable hematoma or history of hemorrhage due to bAVMs, and allowed selective operation;
  2. with recurrent epilepsy seizure, having failed treatment with antiepileptic drugs (AEDs);
  3. with induced deterioration of neurological functions;
  4. With Spetzler-Martin grades from I to IV
  5. Written informed consent is provided by participant in person, or by legal guardians of whom is under 18 years old

### Exclusion criteria

1. > 70 years old with no significant hemorrhagic risk of bAVMs;
2. with Spetzler-Martin Grade ≥ V;
3. accompanied by severe chronic disease, organ dysfunction, or malignant tumor that cannot tolerate the operation;
4. allergic to iodinated contrast agent;
5. refuse to provide written informed consent by participant in person, or by legal guardians of whom is under 18 years old.

### Group assignment and treatment

In the registry set, patients will be assigned to the TO group or the HO group on the basis of clinical conditions and personal wishes. Patients in the HO group will receive microsurgical operation under the assistance of endovascular embolization or balloon occlusion. Patients in the TO group will receive only traditional microsurgical operations, and a repeated DSA will be arranged 3 days after the operation.

In the trial set, patients who refuse the randomized group assignment will be enrolled in the registry set. Those who agree will be randomly (by central randomization system) assigned to the TO group or the HO group. The interventions of each group will be the same in the two sets.

The functional MRI-based neuro-navigation and intraoperative Doppler ultrasound will be used as essential assistance in all operations.

### Outcome evaluation

The primary outcome is defined as neural function deterioration (an increased mRS and mRS > 2) in the 3 months after operation. The neural function will be evaluated 48 h and 7 days after operation while hospitalized and at 3 months, 6 months and 12 months after discharge. A deterioration will be considered temporary if it is sustained < 12 months, otherwise it will be considered permanent. The secondary outcomes will include the postoperative mortality, operation-related complications, and bAVMs residue. The postoperative mortality will include death that occurs within 7 days from the date of operation. Operation-related complications will include any complications that occur within 7 days from the date of operation, including intracranial hemorrhage or infarction, infection of the central nervous system, infection of the respiratory system, cranial nerve deficits, and other symptomatic complications. The bAVMs residue will be diagnosed on the basis of DSA or CTA after operation. The mRS fluctuation will also be recorded and defined as the consecutive changing value of mRS between different follow-up points. The evaluations and diagnoses will be conducted by the physicians who have not learnt about the patients and collected by CRCs.

### Sample size

#### Registry set

According to the relevant literature and data, the average morbidity rate of neural function deterioration is 38.6% [9–11, 34, 35]. The aim of the hybrid operation is to reduce the rate of neural function deterioration by 10%, referring to present studies [31–33]. To ensure the statistical power and the potency of exploring technical details, the sample size is set as the maximum operative capacity of participating centers. According to the records of the latest 5 years, the operative capacity of Beijing Tiantan Hospital is about 220 cases of bAVMs per year, and of other participating centers is 20 cases per year in total. A total of 1200 cases will be recruited into the cohort in the 5-year-recruitment. Among them, 800 cases will receive hybrid operations, and 400 cases will receive microsurgical operations.

#### Trial set

Few studies have been found on bAVMs with perforating feeding arteries. Referring to relevant studies of bAVMs, the neural function deterioration rate was 38.6% in traditional operation and 19% in one-staged hybrid operation. By using PASS 14 (PASS Power Analysis and Sample Size software, NCSS, LLC, Utah, US) with a significance level of 0.05 and a statistical power of 0.9, the minimum sample size for each group is *n* = 117. In total, 236 patients will be enrolled in the trial set.

### Statistical analyses

Statistical analyses will be conducted using the Statistical Package for the Social Sciences software (V.22.0; SPSS, Chicago, Illinois, USA). Demographic information (age, gender, race, provenance, occupation), medical histories (primary symptom, operation history, and past history, family history, smoking and drinking history), bAVM-related morphological and location features (eloquences and fiber tracks involved according to the task), angioarchitectural features (origin and type of feeding arteries, situation of nidus and draining veins), and admitting conditions (signs, mRS score and NIHSS score) will be cataloged into continuous or categorical variables and descriptively analyzed. The baseline conditions of cases involved in the two groups will be compared by the characteristics described above. The Chi-square test and Student t-test will be conducted between homogeneous cases of 2 groups in the outcome variables, including operation-related events (e.g., operative time, hemorrhagic volume, electrophysiological changes) and outcomes to make comparisons of their safety and efficiency in several aspects. Univariate and multivariate logistic regression analyses will be conducted to explore the correlations between the outcome index and variables above. Analyses of subgroups in the hybrid operation group will be performed to evaluate the contributions of different endovascular interventions. In the trial set, differences will be tested with Chi-square test. The effect of hybrid operation in the subgroup will be clarified.

### Data management

All the data in this study will be sourced from experienced physicians and radiologists in 20 participating centers, and they will be prospectively collected by full-time clinical research coordinators (CRCs) using both paper documents and a web-based electronic data capture system (EDC system). SUN HEALTH (Beijing) Health Care Group, a third-party CRO, is contracted to render the services of quality control and data management. The study is supervised and administered by the Ministry of Science and Technology of China and the Beijing Municipal Science and Technology Commission, and it receives resource support from the China National Clinical Research Center for Neurological Diseases. Issues that occur in the cohort will be reported to oversight authorities and evaluated. All data are available only to participating centers while the study is being conducted and will become public once the study concludes. All of the processes of data collection, statistical analysis, study result interpretations and dissemination are under the direct supervision of the principal investigator. The final results will be disseminated via printed media in December 2020.

### Duration of study

The study was approved and authorized to launch in September 2016 and was specified to close in December 2020. The recruitment of participants began on 1st January 2016 in the pilot study that had been launched. Participants enrolled after 1st September 2016 were involved in this study.

## Discussion

The study is designed for a real-world exploration of the benefits and risks of one-staged hybrid operation in the treatment of complex bAVMs. As the interventional clinical study seldom represents an ideal experimental condition, the 2-set design could reduce the study’s compromise of clinical practice. The registry set achieves the coverage of the target population without affecting the normal clinical treatment. General benefits, risks, indications and key technologies could be drawn from it. The trial set focuses on a group of specific participants who would probably get the most benefits from one-staged HO. It could provide high-level evidence as an RCT. The combination of registry and PCT improves the statistical power and research quality of the study in a practical sample size.

With the study, advantages of one-staged hybrid operation will be tested in the treatment of complex brain arteriovenous malformations. Among a variety of therapeutic methods, neither microsurgery, nor endovascular intervention, nor stereotactic radiotherapy could independently meet the requirements of both cure and neural function protection. The multistaged utilization of technical combinations between endovascular embolization and microsurgical resection or stereotactic radiotherapy is available in some bAVMs. Meanwhile, risks exist in the interval period between the stages, especially after partial embolization or stereotactic radiotherapy. The one-staged hybrid operation could eliminate the lesion once and for all without any of those risks. Moreover, it meets the requirement of both cure and neural function protection. However, its benefits have been proposed theoretically with reference to limited attempts [31–33] and experience in cardiac surgery. No high-quality evidence has been acquired to support the proposal. This study is designed to make the comparison between the one-staged hybrid operation and the traditional microsurgical operation for its benefits and risks, as well as the indications. A spanning development of neurosurgical operation might be facilitated by the study, which means a higher cure rate and lower disability rate in patients with complex bAVMs.

### Strengths and limitations

This study, consisting of a prospective real-world registry and a pragmatic clinical trial, will help clarify whether one-staged hybrid operation is a safe and efficient alternative to traditional surgical operation in the treatment of complex bAVMs. It evaluates the effect of one-staged perforating arterial embolization on the surgical resection of complex AVMs. It will help explore indications, key technologies, and workflows of one-staged hybrid operation, as well. However, this study has limitations. Varieties of factors are related to the outcome of complex AVMs. The sample size needs to be enlarged, if further subgroup analyses are required to clarify the effects of factors in details. And there is lower limit of sample size in the trial set. It needs to be critically obeyed to maintain the evidence level of this set.

## Abbreviations

AEDs: Antiepileptic drugs
AKA: As known as
bAVMs: Brain arteriovenous malformations
CRAs: Clinical research associates
CRCs: Clinical research coordinators
CRO: Contract research organization
CT: Computed tomography
CTA: Computed tomographic angiography
Dicom: Digital imaging and communications in medicine
DSA: Digital subtraction angiography
EDC: Electronic data capture system
HO: Hybrid operation
MR: Magnetic resonance
mRS: Modified Rankin Scale
NIHSS: National Institute of Health Stroke Scale
PA: Perforating artery
PCT: Pragmatic clinical trial
RCT: Randomized clinical trial
TO: Traditional operation
